# Exploring the Attribution of Responsibility to Patients Diagnosed With Personality Disorders

**DOI:** 10.1192/bjo.2024.152

**Published:** 2024-08-01

**Authors:** Lauren Glover

**Affiliations:** University of Birmingham, Birmingham, United Kingdom

## Abstract

**Aims:**

This interdisciplinary research explored how responsibility is attributed to patients with personality disorders (PDs). The attribution of responsibility to this group has been extensively discussed by philosophers, and appears to be associated with negative attitudes towards the diagnosis amongst clinicians. This research aimed to both examine the philosophical literature available on this topic, and to explore how future clinicians make judgements of these patients’ responsibility.

A qualitative study was conducted to answer the following four research questions:

1. What do medical students think responsibility means in the context of healthcare?
2. What factors influence when medical students consider patients with mental health disorders, in particular PDs, responsible for their behaviours?
3. How responsible do medical students consider patients with PDs for their behaviours in comparison to patients with other mental health conditions?
4. Do medical students think that responsibility attributions could affect the stigmatisation of the condition and patient care?

**Methods:**

Seven in-depth semi-structured interviews were conducted, involving the discussion of a case report. Interviews had a mean length of 53 minutes. They were then transcribed, coded, and thematic analysis of the data was undertaken. Four main themes were identified: understanding of responsibility, the factors affecting responsibility attribution, stigma and responsibility attribution, and the role of the clinician and the healthcare service.

**Results:**

It was found that medical students considered similar conditions and factors in attributing responsibility to those identified in the philosophical literature. However, several important practical concerns about responsibility attribution in practice were raised, including the possible impact on the therapeutic relationship, difficulties in separating responsibility and blame, and the impact comorbidities and misdiagnoses can have on attributions. Participants believed that stigma towards the diagnosis remains prevalent amongst healthcare professionals, due to stereotypes of these patients being manipulative, and insufficient education about the condition. Additionally, participants highlighted that patient responsibility may be reduced when clinicians and the healthcare service fail to meet their own responsibilities to these patients.

**Conclusion:**

Future research into how other groups of healthcare professionals attribute responsibility is recommended, alongside research into how improved education could reduce stigma and inform responsibility attribution. It is suggested that further education is provided to healthcare professionals about the condition, and more support is offered to those working with patients with PDs to reduce stigma and make the attribution of responsibility fairer to these patients.

